# Ordered GeSi nanorings grown on patterned Si (001) substrates

**DOI:** 10.1186/1556-276X-6-205

**Published:** 2011-03-09

**Authors:** Yingjie Ma, Jian Cui, Yongliang Fan, Zhenyang Zhong, Zuimin Jiang

**Affiliations:** 1 State Key Laboratory of Surface Physics and Department of Physics, Fudan University, Shanghai 200433, People's Republic of China

## Abstract

An easy approach to fabricate ordered pattern using nanosphere lithography and reactive iron etching technology was demonstrated. Long-range ordered GeSi nanorings with 430 nm period were grown on patterned Si (001) substrates by molecular beam epitaxy. The size and shape of rings were closely associated with the size of capped GeSi quantum dots and the Si capping processes. Statistical analysis on the lateral size distribution shows that the high growth temperature and the long-term annealing can improve the uniformity of nanorings.

**PACS** code1·PACS code2·more

**Mathematics Subject Classification (2000) **MSC code1·MSC code2·more

## Introduction

Ordered silicon-based nanostructures have attracted considerable attentions due to their potential applications in various novel devices including field-emission displays [1], nanoelectronic and nanophotonic devices [2-4]. Nanorings are artificial ring-structure in nanoscale that confine carriers in three dimensions. Particularly, they have shown attractive properties due to their special topological configuration, e.g., large and negative excitonic permanent dipole moment [5], memory properties [6] and high oscillator strength for the groundstate band-to-band transition [7]. Self-assembled nanorings [8-11] have been fabricated with a thin capping layer deposited on self-assembled quantum dots (QDs) that were grown by so-called Stranski-Krastanow (SK) growth mode. However, the size uniformity of those nanorings is rather poor and their spatial distribution is random as reported in literatures so far. Nanorings with controllable size and sites have not been reported yet.

In order to obtain nanostructures with an ordered spatial distribution, one promising approach is growing on substrates with ordered nanopattern. Several methods are routinely used to create nanopatterns with controlled size, shape, and spacing, e.g., lithographically induced self-assembly (LISA) [12], holographic lithography [13] and nanoimprint lithography (NIL) [14]. However, those lithographic methods have various deficiencies. The feature sizes of holographic lithography are limited by its interference limit of *λ/*2 and the nanoim-print lithography is a low throughput and high cost technology. Thus, a new kind of nanopattern fabrication method called nanosphere lithography (NSL) [15] has been developed. NSL is an inexpensive, inherently parallel and high-throughput technique. It is capable of producing well-ordered 2 D nanopattern of a wide variety of materials on many substrates. NSL has been used to fabricate periodic GeSi QDs with the period down to 200 nm [16].

It is highly desired to fabricate ordered nanorings in order to study their electronic and magnetic properties. In this study, we explored the growth of long-range ordered GeSi nanorings on patterned Si (001) substrates by molecular beam epitaxy (MBE). Highly ordered inverted pyramid-like pits with {111} facets arranged in a hexagonal lattice on Si (001) substrates were fabricated by NSL and reactive iron etching (RIE) technology. The ordered GeSi nanorings were then grown on those patterned substrates. The size and shape of nanorings were closely associated with the size of capped GeSi QDs and the Si capping processes. Statistical analysis on the lateral size of QDs and nanorings shows that the high growth temperature and the long-term annealing can improve the nanorings' uniformity.

## Experimental

**Figure 1:**
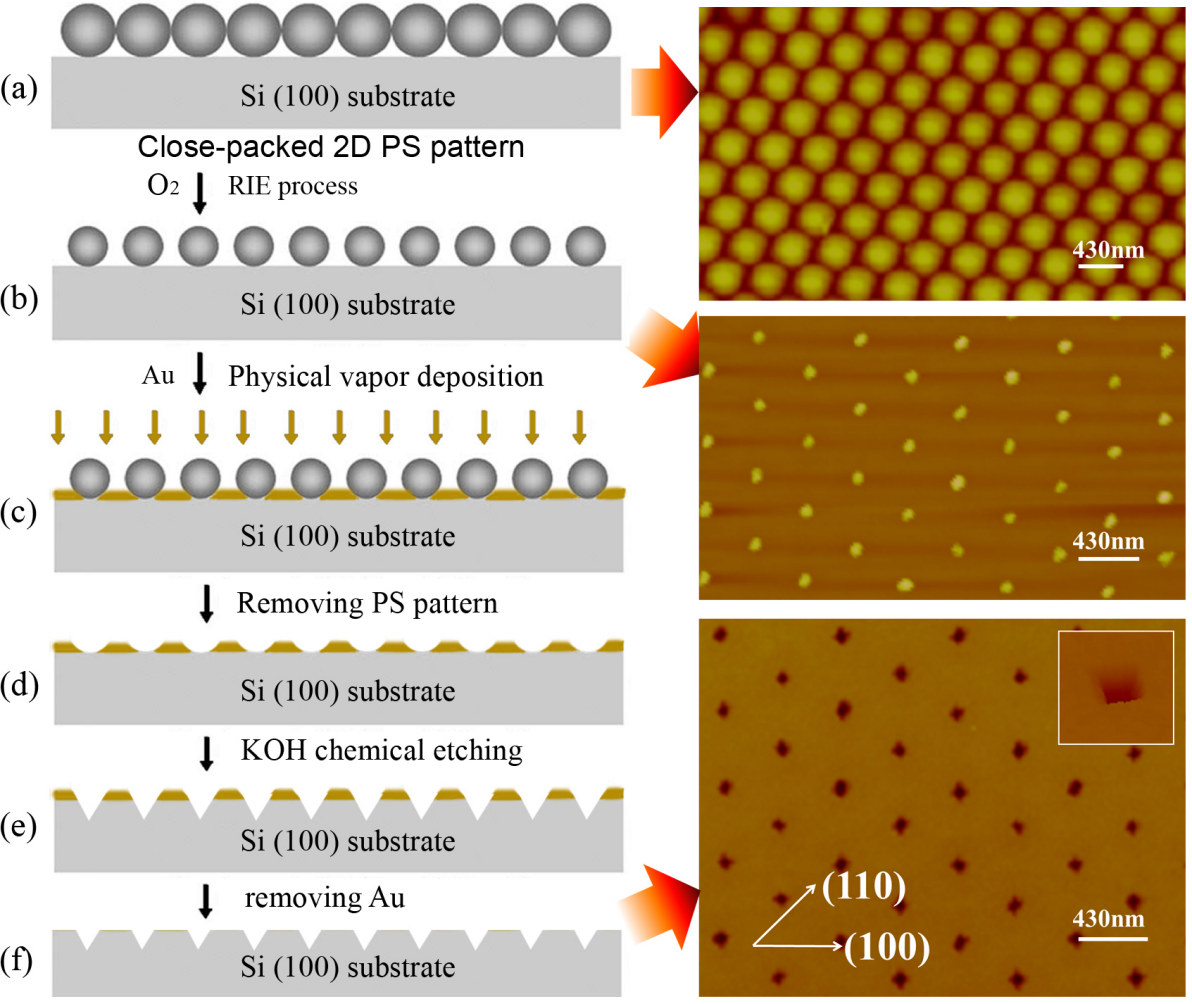
**Schematic illustration for the fabrication of ordered pit-pattern**. **(a) **Closed-packed PS single ML pattern. **(b) **PS pattern after O_2 _RIE. **(c) **Au film deposition. **(d) **Removing PS pattern in THF. **(e) **KOH selective etching. **(f) **Inverted pyramid-like pits pattern with {111} facets after Au was removed. The panels at the right side show the AFM images at corresponding stages.

The schematic illustration for nanopattern fabrication processes is shown in Figure 1. The polystyrene (PS) spheres (Duke Scientific Corporation, Palo Alto, CA, USA) used in this study have a diameter of 430 nm. The PS sphere suspension was diluted by mixing with methanol with a ratio of 1:1. First, the close-packed PS spheres monolayer (ML) was self-assembled on the surface of deionized (DI) water via Weekes' method [17]. The PS ML was then transferred onto a chemically cleaned and hydrogen-terminated surface of p-type Si (001) substrate with a resistivity of 22-32 Ω·cm by draining the DI water, as shown in Figure 1a. Second, the PS spheres ML was etched by RIE to shrink the diameter of PS spheres to about 80 nm (Figure 1b). RIE etching was done using O_2 _(30 sccm) at 30 W, 9.3 Pa for 10 min. Third, a 1 nm thick Au film was deposited onto the surface of the PS covered substrate (Figure 1c). Then the substrate was immersed in tetrahydrofuran (THF) under ultrasonic treatment to remove the PS spheres. A Au-Si alloy and SiO_2 _mask via Au-catalyzed oxidation [18] were left (Figure 1d). The substrate was then etched by KOH solution (20 wt%) at 30°C. With proper etching time, ordered inverted pyramid-like pits with {111} facets were formed, as shown in Figure 1e. The period of the pit-pattern was kept the same as that of the PS pattern, i.e., 430 nm. The average side length and the depth of the square pits was 90 and 40 nm, respectively. The Au and Au-Si alloy mask were removed by immersing the substrate in KI: I_2_: H_2_O (4:1:40) solution for 10 h [19].

**Figure 2:**
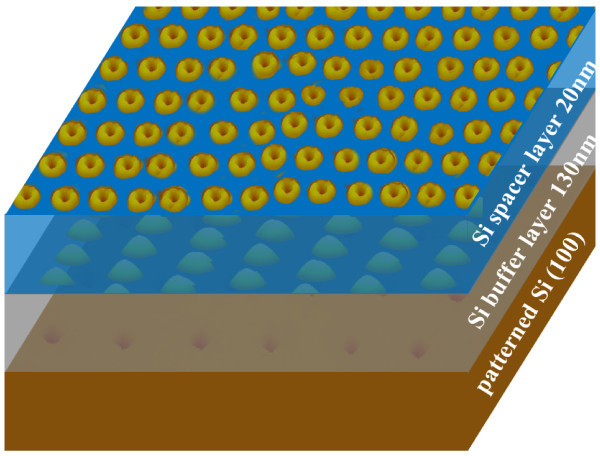
**Schematic sample structure**.

The pit-patterned substrate was^. ^cleaned by RCA method and passivated by HF before loading into the MBE chamber (Riber Eva-32). The growth rate of Si and Ge was 0.5 and 0.06 Å·s^-1^, respectively. The sample structures consist of two layers, one QD layer and one nanoring layer, as illustrated in Figure 2.

**Figure 3:**
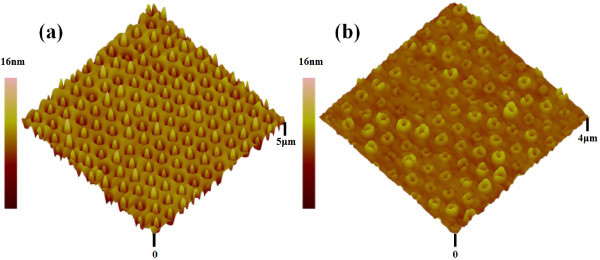
**3D AFM images of (a) ordered GeSi QDs and (b)ordered GeSi nanorings**.

The typical sample growth procedures are as following. After the hydrogen thermal desorption at 860°C for 3 min, a 130 nm thick Si buffer layer was deposited while increasing the growth temperature gradually from 400°C to 500°C to remove surface damages induced by etching [20]. The QD layer was grown by depositing 5 ML Ge while increasing the growth temperature from 500 to 640°C and additional 7 ML Ge at 640°C [21]. The substrate temperature was then decreased to 500°C. A 20 nm thick Si spacer layer was deposited while increasing the growth temperature from 500 to 640°C [22]. To grow nanorings, first, an 8 ML Ge layer was deposited at 640°C to form ordered dome-shaped GeSi QDs, as shown in Figure 3a. Secondly, a 3 nm thick Si capping layer was deposited at the same growth temperature. After growth, the sample was cooled down to room temperature immediately. It was found that the ordered dome-shaped GeSi QDs transformed into ordered GeSi nanorings after the Si capping process, as shown in Figure 3b. The surface morphology of the ordered nanorings was investigated by atomic force microscopy (AFM) (Veeco DI Multimode V SPM and Solver P47-MDT). The post-annealing treatment was done in a high vacuum annealing system (KMT GSL 1600×).

## Results and discussion

**Figure 4:**
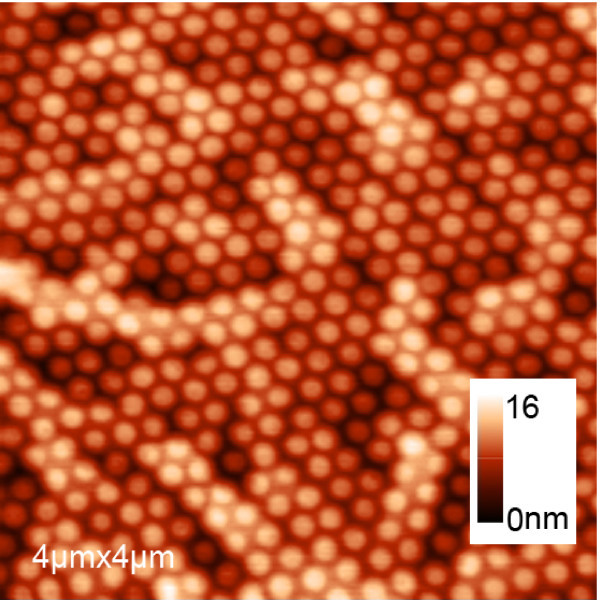
**QDs grown on 200 nm period pit-patterned substrate**. Clearly, GeSi alloy layer between neighboring QDs can be observed.

The distance *d *between neighboring QDs, which is predetermined by the pattern, is crucial for the growth of nanorings on patterned substrates. When *d *is small, e.g., 200 nm, experiment result shows that beside the pits where GeSi QDs are grown, the deposited Ge atoms can also accumulate on the area between neighboring QDs to form a very thin GeSi alloy layer, as shown in Figure 4. A network composed of such thin layers can be seen in a large area AFM scan. In this case, QDs can scarcely transform into nanorings when a thin Si capping layer is deposited, which is similar to the case of very high QD density [23]. By using larger PS nanospheres with a diameter of 430 nm and the fabrication processes described previously, no such network was observed, and most of QDs transformed into nanorings.

To use 430 nm PS spheres, RIE process is necessary to reduce the size of PS spheres to obtain a proper size of pits. When 200 nm PS nanospheres are used, the RIE process is not necessary to modify the PS nanospheres, for the resulting lateral size of the pits is about 100 nm, which is comparable with the size of QDs. However, for 430 nm PS spheres, if no RIE process is performed, after KOH etching, the lateral size of the pits is about 330 nm, which is much larger than that of QDs. When the QDs are grown, more than two QDs may nucleate in one pit, which deteriorates the periodicity. By employing RIE technology, the size of pits is reduced to 92 nm, which insures one QD nucleate in each pit.

**Figure 5:**
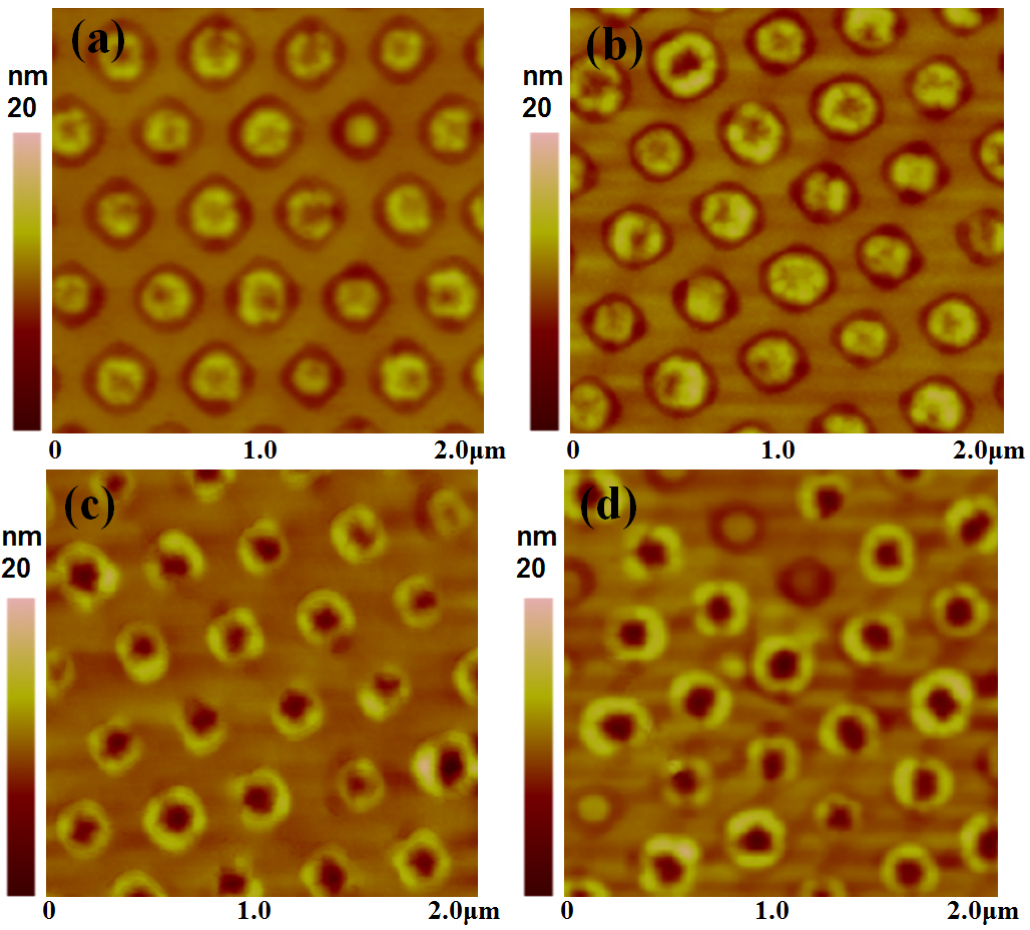
**AFM images of ordered nanorings grown at 610°C with Si capping thicknesses of (a) 1.5 nm (b) 2.0 nm(c) 3.0 nm (d) 4.0 nm**.

Figure 5 shows the AFM images of the samples with different thicknesses of Si capping layers. The periodic characteristic is preserved. At low coverage of Si capping layer (smaller than 2 nm), only shallow dips at the center of QDs can be observed or a portion of QDs transforms into nanorings, as shown in Figure 5a and 5b. When the thickness of Si capping layer is about 3 nm or above, most QDs transform into nanorings, as shown in Figure 5c and 5d. It is clearly seen that this transformation is consistent with the case of flat substrate by Si capping on randomly distributed self-assembled GeSi QDs [10]. The formation mechanism proposed by Cui et al. applies for both flat and patterned substrates.

**Figure 6:**
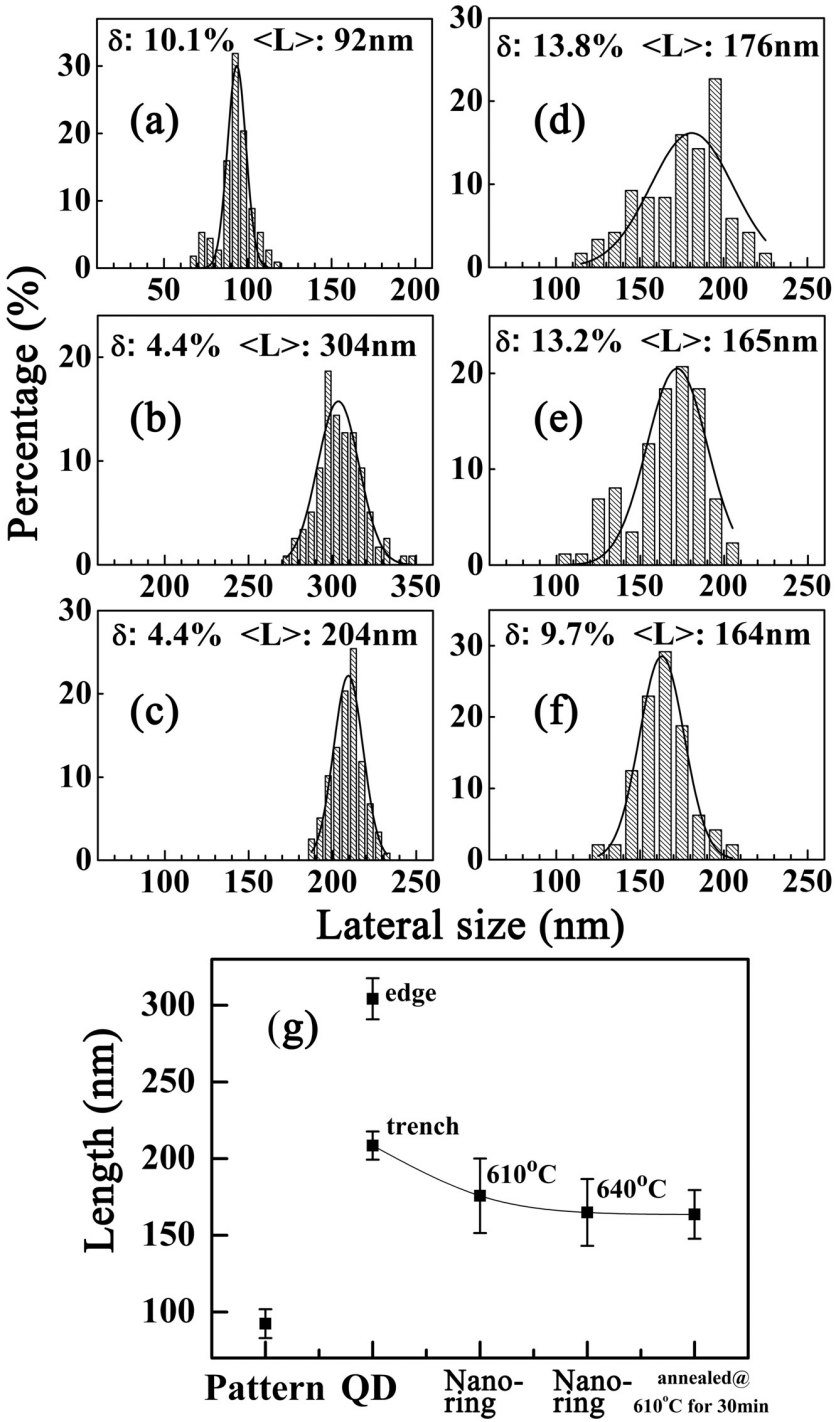
**Distribution of lateral sizes**. **(a) ** pits after etching, **(b) ** pits with QDs therein and diameters of **(c) ** ordered QDs, **(d) ** ordered nanorings grown at 610°C, **(e) ** ordered nanorings grown at 640°C and **(f) ** ordered nanorings grown at 610°C and then annealed at 610°C for 30 min. **(g) **Summary of the mean values.

Figure 6a-f show the histograms of the pit-pattern and the grown samples with different processing parameters. The standard deviation *ΔL*, the mean value 〈*L*〉 and the dispersion *δ *= *ΔL/*〈*L*〉are obtained. All the data can be fitted by a gaussian function, and the fitting curves are also given. The mean values are plotted in Figure 6g. The mean lateral size of the fabricated pits is about 92 nm, which is even smaller than that fabricated by using 200 nm PS nanospheres. However, *δ *is as large as 10%, which is caused by the RIE process, because *δ *of 430 nm PS nanospheres is only about 3% and KOH etching process has slight influences [16]. From the AFM images of ordered QDs and nanorings, deep trenches, which often exist in QDs grown on patterned substrate [13], can be observed around QDs and nanorings. For the ordered QD sample, each QD locates in one pit. The mean lateral size of the pits is 304 nm, which is much larger than that of the original pits. The enlargement of the lateral size of the pits may be caused by the anisotropically grown buffer layer.

**Figure 7:**
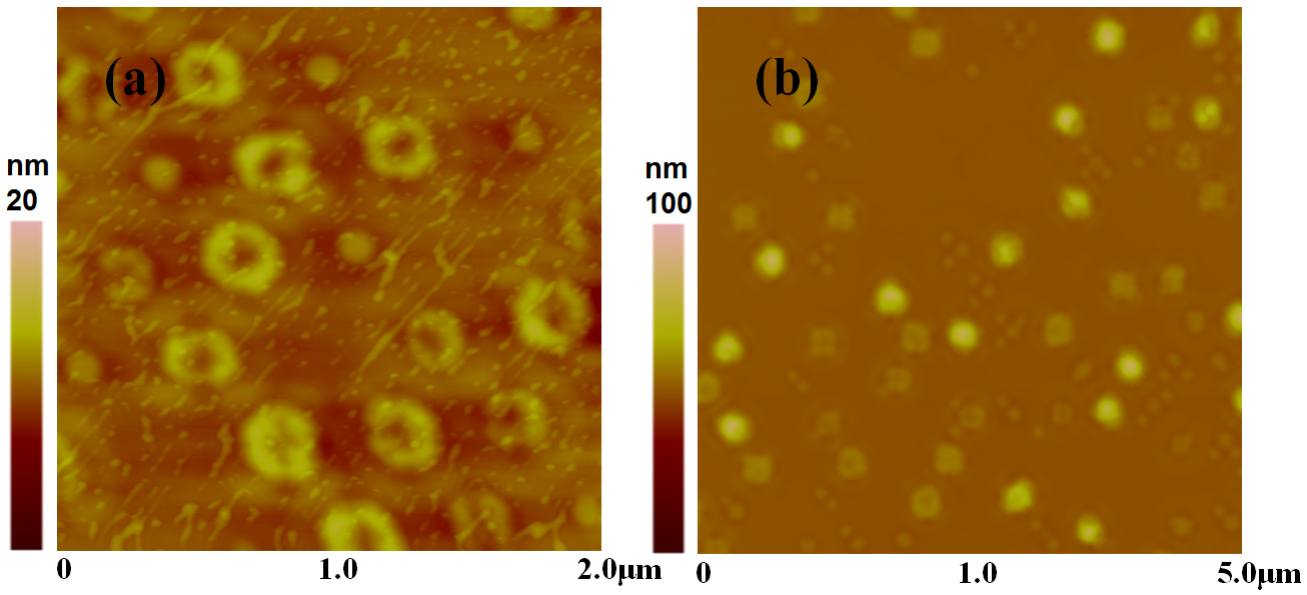
**AFM surface morphologies of ordered nanorings with 2 nm Si capping**. **(a) **Post-annealing for 0.5 h at 610°C. **(b) **Post-annealing for 1 h at 610°C.

By Si capping at 610°C, 〈*L *〉of transformed nanor-ings is 175 nm, which is relatively smaller than that of QDs (208 nm). 〈*L*〉 decreases to 165 nm when the growth temperature of Si capping increases to 640°C. *ΔL *also decreases from 24.3 to 21.8 nm. Long-term annealing can help the transformation from QDs to nanorings [24]. By annealing the sample shown in Figure 5b at 610°C for 30 min in high vacuum (10^-7 ^Torr), we found that 32% of QDs were converted into nanorings, as shown in Figure 7a. The other QDs disappeared by mass migration and no trenches were observed around the nanorings. The mean lateral size (163 nm) is close to that by capping at 640°C. *ΔL *is as small as 15.9 nm. It can be seen that both high growth temperature and long-term annealing can improve the size uniformity. However, if the annealing time was extended to 60 min, the mass migration and SiGe intermixing effects resulted in the appearance of super domes, as shown in Figure 7b. In this case, no nanorings existed any more.

## Conclusion

In summary, we proposed an approach to fabricate highly ordered nanorings with controllable period by NSL and RIE technology. Ordered 430 nm period GeSi nanorings were successfully fabricated on the ordered pit-patterned Si (001) substrates. The size and shape of rings were closely associated with the size of capped GeSi QDs and the Si capping process. Statistical analysis on the lateral size distribution shows that the high growth temperature and the long-term annealing can improve the nanorings uniformity.

## Abbreviations

AFM: atomic force microscopy; DI: deionized; LISA: lithographically induced self-assembly; ML: monolayer; NIL: nanoimprint lithography; NSL: nanosphere lithography; QDs: quantum dots; RIE: reactive iron etching; SK: Stranski-Krastanow; THF tetrahydrofuran.

## Competing interests

The authors declare that they have no competing interests.

## Authors' contributions

MYJ, CJ conceived and designed the experiments. MYJ carried out the experiments with contribution from CJ, FYL, ZZ and JZM. CJ and ZZ supervised the work. MYJ, CJ wrote the manuscript. All authors read and approved the final manuscript.
